# Intergenerational Transmission of Proactive Health Behaviors Among Adolescents with Overweight or Obesity: The Mediating Role of Self-Efficacy and Family Cohesion

**DOI:** 10.3390/nu17213377

**Published:** 2025-10-28

**Authors:** Tian Hu, Jingwei Zhou, Lianlong Yu, Suyun Li, Qian Ning Leong, Jingjing Li, Yunping Zhou, Ying Jiang

**Affiliations:** 1 School of Nursing, Qingdao University, 308 Ningxia Road, Qingdao 266071, China; 2 Qingdao Municipal Key Laboratory for Smart Healthcare and Chronic Disease Management, Qingdao 266071, China; 3 Shandong Center for Disease Control and Prevention, 16992 Jingshi Road, Jinan 250014, China; 4 School of Nursing, National University of Singapore, Singapore 117597, Singapore

**Keywords:** adolescents, obesity, proactive health behaviors, intergenerational transmission, self-efficacy, family cohesion

## Abstract

**Background/Objectives:** The family environment exerts a significant influence on the development of weight-related behaviors among adolescents. However, the lack of motivation often leads to failure in sustaining health behaviors, particularly among adolescents with overweight and obesity. This study examined whether parental healthy behaviors are associated with proactive health behaviors among adolescents with overweight and obesity and investigated the mediating roles of adolescents’ self-efficacy and family cohesion in these relationships within Chinese families. **Methods**: We recruited 4932 adolescents with overweight and obesity aged 10 to 15 in Shandong Province between October and December 2024. Participants completed validated questionnaires assessing parental healthy behaviors, self-efficacy, family cohesion, and proactive health behaviors. We analyzed the data using multilevel regression models and mediation analysis, controlling for key sociodemographic factors. Subgroup analyses were conducted by gender and region level. **Results**: Both fathers’ (β = 0.442, 95% CI = 0.263, 0.620) and mothers’ (β = 0.525, 95% CI = 0.336, 0.714) healthy behaviors were positively associated with proactive health behaviors among adolescents with overweight and obesity in the adjusted model. Further mediation analysis revealed that adolescent self-efficacy and family cohesion significantly mediated these relationships. **Conclusions**: This work suggested that parental healthy behaviors were positively associated with proactive health behaviors among adolescents with overweight and obesity. Additionally, higher self-efficacy and family cohesion enhance these associations. The findings offered evidence-based recommendations for creating family-centered interventions targeting adolescents with overweight and obesity. Future research should employ longitudinal designs to understand these relationships better and improve the efficacy of family-based interventions for adolescents with overweight and obesity.

## 1. Introduction

Obesity, a lifestyle-related disorder, has surged to epidemic levels and now ranks among the foremost public health challenges [1]. In addition to its short-term effects in adolescence, obesity has the potential to have long-term adverse impacts on health [2]. Compared with peers who maintain a healthy weight, adolescents with obesity are more likely to remain obese into adulthood [3]. Unhealthy behaviors are considered some of the main underlying causes of overweight and obesity [4]. Consequently, health behavior interventions are central to the effective management of adolescents with overweight and obesity [5]. The family environment significantly influences the development of weight-related behaviors among adolescents, such as physical activity, dietary behaviors, and sedentary behaviors [6,7]. However, a lack of motivation often leads to failure to sustain health behaviors, particularly among adolescents with overweight and obesity [8,9]. This highlights the importance of adolescents’ proactive health behaviors in weight management.

The definition of proactive health behaviors is an individual’s ability to proactively acquire health information and engage in health behaviors, which reflect their motivation and willingness to adopt and maintain good health [10]. Proactive health behaviors encompass not only the adoption of healthy behaviors but also the self-directed maintenance of these behaviors over time, which is particularly crucial for adolescents with overweight and obesity [8]. In recent years, proactive health has garnered increasing attention from researchers. A meta-analysis suggested that autonomous motivation serves as a valid focus for interventions aimed at promoting health behavior change [11]. Supportive family environments, particularly those established by parents, play a crucial role in fostering proactive health awareness and promoting health behavior changes among adolescents [12]. However, a critical gap remains in understanding the underlying mechanisms of the intergenerational transmission of proactive health behaviors and how family characteristics moderate these associations in adolescents with overweight and obesity. Therefore, further exploration of how family characteristics contribute to proactive health behaviors is essential for developing family-centered intervention strategies for adolescents with overweight and obesity.

Following the introduction of the health-promoting family conceptual model by Christensen, extensive research has been published, including both theoretical discussions and empirical investigations based on this framework [13]. Among these efforts, the German scholar Niermann focused on the internal dynamics of family microsystems and developed the Levels of Interacting Family Environmental Subsystems (LIFES) framework [14]. Previous studies have described the importance of context in determining healthy behaviors and the effectiveness of interventions designed to promote healthy behaviors. As a context closely connected to adolescents, the family exerts a profound and lasting influence on their physical and mental health, as well as their health behaviors [15]. According to the LIFES framework, the internal family environment is categorized into three subsystems: the individual system, the parent-child system, and the family system. On the basis of previous research and the LIFES framework, parental health behaviors (individual system), self-efficacy (individual system), and family cohesion (parent-child system) were selected as the representative variables.

It has long been recognized that there is a strong association between the health behaviors of parents and those of their offspring, even if the offspring are not consciously aware of the influence their parents have on them [16]. The intergenerational transmission of health behaviors can occur through direct social learning from parents [17]. A prospective cohort study reveals that adherence to healthier parental behaviors is associated with a substantially lower risk of obesity in adolescence [18]. Studies from the UK and Ireland suggest that parental dietary behaviors influence the intergenerational transmission of overweight and obesity [19,20]. Obesogenic behaviors exhibited by parents, such as unhealthy diets, physical inactivity, and sedentary behaviors, may lead adolescents with overweight and obesity to adopt the same behaviors [21]. A survey of parent-adolescent dyads indicated that the frequency of parents’ fruit and vegetable intake was significantly correlated with adolescents’ proactive health motivation and served as a predictor of their fruit and vegetable intake frequency [12].

The concept of self-efficacy, introduced by A. Bandura in 1977, refers to an individual’s belief in their ability to perform a task successfully [22]. This belief is closely linked to positive outcomes during adolescence. Research has shown that parents’ health behaviors and social control significantly influence adolescents’ self-efficacy and health behaviors [23]. Parental support and adolescents’ personal mastery experiences within the family are particularly influential in the development of self-efficacy [24]. Additionally, a lifestyle intervention utilizing mobile technology suggests that self-efficacy plays a crucial role in modifying eating behaviors and increasing physical activity levels [25]. Similarly, a survey conducted among 400 Chinese middle school students indicates that interventions aimed at promoting physical activity should prioritize enhancing students’ self-efficacy [26]. Therefore, it is reasonable to conclude that parental healthy behaviors are positively associated with higher self-efficacy in adolescents with overweight and obesity, which in turn influences their choice to engage in proactive health behaviors.

Family cohesion refers to the emotional bonds shared among family members, and is characterized by a sense of togetherness and support [27]. When parents engage in healthy behaviors, family members are more likely to adopt similar lifestyles, thereby enhancing family cohesion and identification. Conversely, family conflicts arising from parents’ unhealthy behaviors, such as smoking and excessive drinking, can undermine family harmony and cohesion. Given that family cohesion plays an important role in adolescents’ self-discipline, it can influence their engagement in binge eating behavior, drinking, and other obesogenic behaviors [28,29]. Several studies strongly support the positive association between family cohesion and the physical and mental health of family members [30]. Therefore, it is reasonable to propose that healthy behaviors adopted by parents are positively associated with family cohesion, which in turn fosters proactive health behaviors among adolescents with overweight and obesity.

In conclusion, parental healthy behaviors, adolescent self-efficacy, and family cohesion correlate with proactive health behaviors among adolescents with overweight and obesity. Therefore, this study proposes two hypotheses, as illustrated in Figure S1.

**H1.**  *Self-efficacy mediates the association between parental healthy behaviors and proactive health behaviors among adolescents with overweight and obesity.*

**H2.**  *Family cohesion mediates the association between parental healthy behaviors and proactive health behaviors among adolescents with overweight and obesity.*

## 2. Materials and Methods

### 2.1. Study Design and Participants

This cross-sectional study was conducted in Shandong Province, China, from October to December 2024. The project employed a stratified cluster random sampling method. Three survey cities were selected on the basis of geographical location, eating habits, and economic characteristics. The schools were randomly sampled, and classes were selected randomly according to the grade distribution, with each class serving as a unit. All the students from the selected classes were invited to participate and a total of 18,328 students from 87 schools participated in this survey. Adolescents with overweight or obesity were identified using age-sex-specific percentile curves of body mass index (BMI) [31] and were invited to participate in this research. Adolescents who were absent from school, had serious physical or mental health conditions, did not live with both parents, or had incomplete questionnaires were excluded from the analysis. Totally, the current research comprised 4932 students aged 10–15 years who were classified as overweight or obese. All participants were informed of the study’s purpose and received standardized instructions for completing the questionnaires. Written informed consent was obtained from all participants or their parents or guardians prior to the survey. This study was conducted in accordance with the Declaration of Helsinki. The Ethics Committee of the Medical College of Qingdao University approved the study (QDU-HEC-2024567).

### 2.2. Measure

#### 2.2.1. Parental Healthy Behaviors

Parental healthy behaviors were assessed using 14 questions related to exercise, balanced diet, smoking, and alcohol consumption [32] (Table S1). Exercise was evaluated through three positive indicators. The assessment of a balanced diet included the intake of fruits and vegetables. In the questionnaires, exercise and balanced diet were scored on a 4-point Likert scale ranging from 1 (“Never”) to 4 (“Every day”). Smoking was measured using binary variables. Alcohol consumption was assessed on the basis of alcohol consumption frequency (more than twice a week). The Cronbach’s α coefficient for this study was 0.867.

#### 2.2.2. General Self-Efficacy

The General Self-Efficacy (GESE) Scale was developed by Schwarzer in 1981 and has been widely used worldwide. The reliability and validity of the Chinese version of the GESE scale have been verified by Wang [33]. The GESE scale was a unidimensional scale with 10 items, all of which were rated on a 4-point Likert scale. For example, the response options for the item “I can always manage to solve difficult problems if I try hard enough” ranged from “not at all true” to “exactly true” with scores assigned from 1 to 4. The total score on the scale ranges from 10 to 40. Higher scores indicate a greater sense of self-efficacy. In this study, the Cronbach’s α coefficient for the GESE in this study was 0.938.

#### 2.2.3. Family Cohesion

The Family Adaptability and Cohesion Evaluation Scales (FACESs) were developed by Olson in 1982, and the Chinese version was translated and adapted by Lipeng Fei [34]. This scale consists of two dimensions: family cohesion and family adaptability. Family cohesion refers to the degree of emotional connection among family members. This study focused on the family cohesion evaluation subscale (Table S2), which is suitable within the Chinese context and has good validity [35]. The subscale includes 16 items, with scores ranging from 1 to 5 for each item. A sample item is “We share interests and hobbies with each other”. The total score ranges from 16 to 80, with higher scores indicating greater family cohesion. The Cronbach’s α coefficient was 0.887 in this study.

#### 2.2.4. Proactive Health Behaviors

The Proactive Health Behaviors Scale was developed by Fan in 2024 and demonstrated good reliability and validity [36] (Table S3). This scale provides a tool for measuring proactive health behaviors among adolescents in China. The self-report questionnaire encompasses dimensions of health responsibility, physical activity, nutritional diet, mental health, and self-discipline. It consists of 24 items and is scored on a 5-point Likert scale ranging from 1 (“never”) to 5 (“always”). A sample item is “I proactively acquire knowledge and information about health management”. The total score ranges from 24 to 120, with greater scores indicating higher levels of proactive health behaviors. The Cronbach’s α coefficient was 0.956 in this study.

#### 2.2.5. Covariates

Several sociodemographic factors were considered confounders: individual system (adolescents’ age, gender, grade, ethnicity [Han or minority], accommodation type [boarding or nonboarding], and parental educational attainment) and family system (only child, family economic status, region [urban or rural], and area [coastal or inland]). Parental educational attainment was reported on a scale ranging from 1 (primary school or below) to 4 (college or above). Family economic status was self-assessed by the participants and categorized into three levels.

### 2.3. Statistical Analysis

STATA (Version 17.0) and IBM SPSS Statistics (Version 25.0) were utilized for all the data analyses. The Kolmogorov-Smirnov test was used to assess the normality of continuous variables. The medians and interquartile range (Q1–Q3) were used to describe categorical variables. Frequencies and percentages were used to describe categorical variables. The Wilcoxon rank-sum (Mann–Whitney) test and Kruskal–Wallis test were used to assess differences in the demographic characteristics of proactive health behaviors. We performed multiple linear regression analyses to estimate the associations between family variables and proactive health behaviors. To explore the independent associations of the three systems, sociodemographic confounding factors for the subsystems were sequentially included in the adjusted model. Subsequently, Spearman correlation analyses were used to estimate the relationships among parental healthy behaviors, self-efficacy, family cohesion, and proactive health behaviors. Finally, we tested whether self-efficacy and family cohesion mediated the associations between parental healthy behaviors and proactive health behaviors. Additionally, to identify any differences in the results, we conducted subgroup analyses on the basis of gender and region. To enhance the robustness and generalizability of our findings, we conducted supplementary analyses comparing adolescents with overweight and obesity to those with underweight and normal-weight in terms of the influence of parental healthy behaviors.

## 3. Results

Our analysis included 4932 adolescents with overweight and obesity, and their sociodemographic information was presented in Table 1. The prevalence of overweight and obesity was higher among males (3124, 63.34%) compared with females (1808, 37.66%). The distribution of educational attainment among fathers was similar to that among mothers, with approximately a quarter having completed a college degree or higher. Over two-thirds of the participants lived in multiple-child households (62.96%) or lived in urban areas (60.75%). Adolescents with overweight and obesity who were male, boarding, with higher parental education attainment and family economic status had higher scores on proactive health behaviors.

Table 2 presented the results of multilevel models that examine the associations between parental health behaviors and proactive health behaviors among adolescents with overweight and obesity, while controlling for various sociodemographic confounding factors across different subsystems. In the unadjusted model, a statistically significant association was found between parental healthy behaviors and proactive healthy behaviors among adolescents with overweight and obesity (fathers’ healthy behaviors: β = 1.090, 95% CI = 0.889, 1.291; mothers’ healthy behaviors: β = 0.845, 95% CI = 0.629, 1.061). In the adjusted analyses, Model 2 incorporates additional variables from the individual system; Model 3 adds variables from the parent–child system; and Model 4 adds variables from the family system. The associations between parental healthy behaviors and proactive healthy behaviors among adolescents with overweight and obesity remained significant across Model 2, Model 3, and Model 4. Proactive health behaviors among adolescents with overweight and obesity were positively associated with increased self-efficacy, healthier parental behaviors, stronger family cohesion, and better family economic status. Additionally, males with overweight and obesity were more likely to engage in proactive health behaviors compared to females with overweight and obesity (β = 1.184, 95% CI = 0.226, 2.142). Adolescents with overweight and obesity residing in rural and coastal areas exhibited a higher level of proactive health behaviors.

In the subgroup analyses, the associations between parental healthy behaviors and proactive health behaviors among adolescents with overweight and obesity remained significant (Tables S4 and S5). In the gender-specific analysis, a stronger relationship was observed between fathers’ healthy behaviors and proactive health behaviors in males (β = 0.551, 95% CI = 0.321, 0.781, *p* < 0.001) than in females (β = 0.284, 95% CI = 0.002, 0.567, *p* = 0.049). Conversely, a stronger relationship was found between mothers’ healthy behaviors and proactive health behaviors in female (β = 0.758, 95% CI = 0.457, 1.059, *p* < 0.001) than in boys (β = 0.380, 95% CI = 0.137, 0.624, *p* = 0.002). In the region-specific analysis, the associations between mothers’ healthy behaviors and proactive health behaviors among adolescents with overweight and obesity were not statistically significant in rural areas (β = 0.281, 95% CI = −0.035, 0.598, *p* = 0.082). In supplementary analyses (Table S6), mothers’ healthy behaviors showed a stronger association with adolescents’ proactive health behaviors in the underweight and normal-weight group (β = 0.723, 95% CI: 0.567–0.880) compared to the overweight and obesity group (β = 0.525, 95% CI: 0.336–0.714).

The bivariate correlations for the main study variables are presented in Table S7. All variables remained significantly correlated after applying Bonferroni correction. The results indicated that fathers’ healthy behaviors were positively related to self-efficacy (β = 0.228, *p* < 0.001), family cohesion (β = 0.314, *p* < 0.001), and proactive healthy behaviors (β = 0.335, *p* < 0.001). Similarly, mothers’ healthy behaviors were also positively related to self-efficacy (β = 0.230, *p* < 0.001), family cohesion (β = 0.290, *p* < 0.001), and proactive healthy behaviors (β = 0.330, *p* < 0.001). Additionally, self-efficacy and family cohesion were positively related to proactive healthy behaviors.

**Figure 1:**
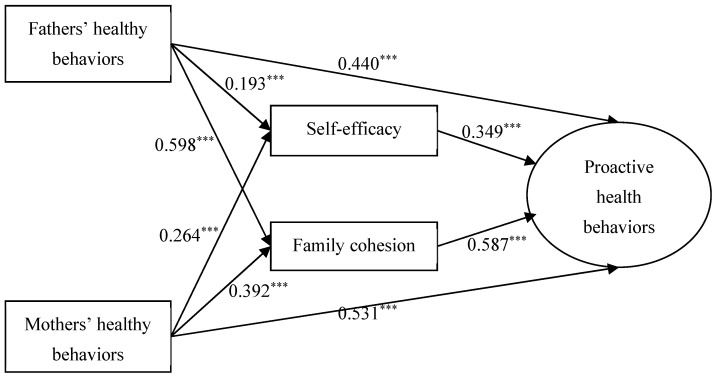
Mediation analysis of self-efficacy and family cohesion in the relationship between parental healthy behaviors and overweight/obese adolescents’ proactive health behaviors. Note: *** *p* < 0.001.

As shown in Table 3 and Figure 1, the direct effect of fathers’ healthy behaviors on proactive healthy behaviors among adolescents with overweight and obesity was significant (direct effect = 0.553, 95% CI = 0.373, 0.734, *p* < 0.001). Moreover, fathers’ healthy behaviors were associated with proactive healthy behaviors through the mediating roles of self-efficacy (indirect effect = 0.096, 95% CI = 0.011, 0.027, *p* < 0.001) and family cohesion (indirect effect = 0.441, 95% CI = 0.070, 0.105, *p* < 0.001). In addition, the total effect of fathers’ healthy behaviors on proactive healthy behaviors among adolescents with overweight and obesity was also significant (total effect = 1.090, 95% CI = 0.889, 1.291, *p* < 0.001).

In addition, the direct effect of mothers’ healthy behaviors on proactive healthy behaviors among adolescents with overweight and obesity was also significant (direct effect = 0.498, 95% CI = 0.305, 0.690, *p* < 0.001). Mothers’ healthy behaviors indirectly affected proactive healthy behaviors through self-efficacy (indirect effect = 0.101, 95% CI = 0.011, 0.027, *p* < 0.001) and family cohesion (indirect effect = 0.246, 95% CI = 0.031, 0.061, *p* < 0.001). The total effect of mothers’ healthy behaviors on proactive healthy behaviors among adolescents with overweight and obesity was significant (total effect = 0.845, 95% CI = 0.629, 1.061, *p* < 0.001).

## 4. Discussion

Guided by the LIFES framework, the present study examined the intergenerational transmission of proactive healthy behaviors among adolescents with overweight and obesity and further tested the mediating role of self-efficacy and family cohesion in Chinese families. Our findings demonstrated that proactive healthy behaviors among adolescents with overweight and obesity were transmitted across generations from both fathers’ and mothers’ healthy behaviors. In addition, our study revealed that self-efficacy and family cohesion partially mediated the intergenerational transmission of parental healthy behaviors to proactive healthy behaviors among adolescents with overweight and obesity.

Our findings found that the variables in the individual system, the parent-child system, and the family system were collectively associated with proactive healthy behaviors among adolescents with overweight and obesity. This finding is consistent with those of previous studies. A randomized clinical trial in the U.S. suggested that family has an important and modifiable influence on the development of obesity among adolescents, and adolescents would imitate the healthy behaviors of parents or siblings, such as developing healthy eating and physical activity [37]. However, in our study, having siblings was not significantly associated with proactive health behaviors among adolescents with overweight and obesity. According to resource dilution theory, offspring may benefit from being an only child, which counteracts the influence of the lack of interaction among siblings to a certain extent [38]. In a qualitative study of triadic interactions between adolescents, caregivers, and health-care providers, researchers reported that promoting and building adolescent initiative was the focus of helping adolescents to choose healthier behaviors [39]. Therefore, both proactivity and family environments are essential for effective weight management and the development of proactive healthy behaviors in adolescents with overweight and obesity.

Furthermore, we found that males were more likely to maintain proactive healthy behaviors. This may be explained by different societal expectations [40] and different senses of family pressure and support [41]. On one hand, societal norms impose a more pronounced “athletic expectation” upon males: physical activity is broadly perceived as an essential element of masculinity [42]. There are more exercise opportunities and positive feedback for males to be active at school and in the community, whereas there may be fewer opportunities that meet the needs and interests of females [43]. On the other hand, parents typically tend to offer males more active encouragement and resources (e.g., sports clubs) while frequently commending their health-promoting endeavors. In contrast, parental attention toward females tends to focus more on academic achievement than on improving athletic skills. These differences may endow males with an enhanced perception of self-efficacy and increased external validation in the context of developing proactive healthy behaviors. Future interventions should focus on creating more appealing activities and fostering a more supportive family atmosphere for females to narrow the gender gap.

This study found that mothers’ healthy behaviors showed a stronger association with adolescents’ proactive health behaviors in the underweight and normal-weight group compared to the overweight and obesity group. Mother often play a significant role in managing the daily care and health of family members, such as creating a healthy dietary environment [44], which exerts a significant impact on adolescents’ proactive health behaviors. However, for adolescents with overweight and obesity, school-based prevention efforts can help reduce barriers to healthy eating and support the adoption of healthy lifestyles [45]. Peer intervention among adolescents with overweight and obesity has also demonstrated strong evidence of effectiveness, especially in promoting physical activity [46]. Additionally, community engagement interventions that involve environmental changes, behavioral modifications, or a combination of both are feasible methods for addressing obesity in adolescents [47]. All these factors may potentially weaken the association between mothers’ healthy behaviors and proactive health behaviors among adolescents with overweight and obesity. By simultaneously considering the influences of both fathers’ and mothers’ behaviors on adolescents’ proactive healthy behaviors in the same models, we found that parental healthy behaviors were transmitted across generations to proactive healthy behaviors among adolescents with overweight and obesity. Drawing on the past literature, we propose three possible explanations for this intergenerational transmission. First, according to social learning strategies, people can form preferences by observing others’ behaviors and outcomes [48]. Adolescents tend to imitate their parents’ behavior, and parental healthy behaviors are integrated into the family’s daily life. A systematic review showed that parent-based interventions could improve health behaviors among adolescents [49]. Second, parents with strong health consciousness usually provide their children with more health resources, such as better diet quality [50]. A previous study revealed significant associations between high diet quality scores and obesity measures [51]. Adolescents’ healthy behaviors are rooted in parents’ expansive understanding of health [52], which shapes their knowledge of and attitude toward obesity management. Third, parents with better economic status or higher educational attainment levels are more capable of providing material or psychological support to foster proactive healthy behaviors among adolescents with overweight and obesity [53]. Both healthy behaviors and socioeconomic advantages are increasingly transmitted in families [54]. In conclusion, promoting proactive health behaviors through parents is critical for maintaining long-term well-being among adolescents with overweight and obesity.

We examined the mediating effects of self-efficacy on the relationships between parental healthy behaviors and proactive health behaviors among adolescents with overweight and obesity. Our findings were supported by several past studies. Nakamura et al. demonstrated that parental healthy behaviors and social control could influence adolescents’ self-efficacy and health behaviors [23]. This aligns with our study, suggesting that parental healthy behaviors may serve as both a direct model and an indirect facilitator through self-efficacy, thereby improving proactive health behaviors and obesity management in adolescents with overweight and obesity. A meta-analysis indicated a significant positive association between self-efficacy and proactive health behaviors among adolescents [55]. Multiple studies reinforce the reliability of our findings. Self-efficacy can influence individuals’ behaviors on the basis of their beliefs, thereby enhancing their capacity to initiate and maintain healthy behaviors, regardless of external and internal factors [56]. In a study that utilized actor-partner interdependence model analyses involving 1854 parent-adolescent dyads, the correlation between self-efficacy and physical activity was found to be the strongest [57]. These previous studies strengthen the validity of our findings and suggest that self-efficacy plays a crucial role in the intergenerational transmission of parental healthy behaviors to proactive health behaviors among adolescents with overweight and obesity.

Our results indicated that family cohesion also plays a mediating role in the relationship between parental healthy behaviors and proactive health behaviors among adolescents with overweight and obesity. A longitudinal study spanning 16 waves revealed that family cohesion could enhance healthy behaviors in both parents and children [58]. This finding is consistent with our results, indicating that family cohesion fosters an environment conducive to the development of proactive health behaviors and weight control during adolescence. Additionally, several studies had shown that family cohesion could mitigate the negative impacts of parental alcohol abuse on adolescents [59]. These studies indicated that the protective role of family cohesion is particularly significant in difficult family situations. Quick [60] revealed that offspring’s mental and physical health, as well as their physical activity levels were better in families with greater family social capital, which includes supportive relationships, family cohesion, and lower levels of family conflict. A national cross-sectional study involving 2379 girls indicated that enhancing family cohesion could be an important objective for encouraging healthy behavior, such as increasing breakfast consumption and reducing soda intake, which could help in managing obesity [61]. Nevertheless, a study focusing on a sample of Latines reported that the interaction between family cohesion and acculturative stress was not significantly associated with binge eating [62]. This finding indicates that the effectiveness of family cohesion may be moderated by specific cultural factors, such as experiences of discrimination, highlighting the importance of considering contextual factors when devising weight-loss programs.

This study is the first to examine the relationships between parental healthy behaviors, adolescents’ self-efficacy, family cohesion and proactive health behaviors focused exclusively on adolescents with overweight and obesity. Additionally, we included a relatively large sample size to ensure better representation. However, it is important to acknowledge the limitations of this study. First, owing to the cross-sectional study design, it is difficult to make inferences about causality. For instance, observed associations may be bidirectional, as parents and adolescents continuously influence and reshape each other. To gain a deeper understanding of temporal dynamics and potential causality, longitudinal studies are essential. Second, the sample was limited to Shandong Province. Therefore, it remains to be considered when generalizing the findings to other regions. Third, it is also useful to include additional variables in the analysis, such as the weight status of the parents. Future research should consider these variables and further investigate their role in the intergenerational transmission of proactive health behaviors among adolescents with overweight and obesity. Finally, although self-report tools with high reliability and validity were utilized, participant responses may have been compromised by recall bias and weight stigma. In our study, the information was collected anonymously and kept confidential, which may have helped reduce the bias associated with weight stigma. Future research should consider collecting data from multiple sources or employ objective measurements.

## 5. Conclusions

This study demonstrated that parental healthy behaviors promoted proactive healthy behaviors among adolescents with overweight and obesity. Moreover, the results also indicated that self-efficacy and family cohesion could serve as partial mediators in the association between parental healthy behaviors and proactive healthy behaviors among adolescents with overweight and obesity. This study enhances our understanding of how families influence proactive health behaviors and offers evidence-based recommendations for creating family-centered interventions targeting adolescents with overweight and obesity. Future studies should utilize longitudinal methods to explore the lasting effects of families on proactive healthy behaviors among adolescents with overweight and obesity and reveal how family dynamics influence obesity management across different stages of adolescent development.

## Figures and Tables

**Table 1:** Socio-demographic information of the participants (*N* = 4932).

Variables	*N* (%)	Proactive Health Behaviors Scores (M ± SD)	Z/H	*p*
Age	4932	86.50 ± 18.78	18.20 ^b^	0.003
Gender			−4.63 ^a^	<0.001
Female	1808 (37.66)	88.36 ± 19.53		
Male	3124 (63.34)	90.82 ± 20.33		
Ethnicity			−0.68 ^a^	0.500
Minority	87 (1.76)	88.23 ± 20.84		
Han	4845 (98.24)	89.95 ± 20.07		
Accommodation			2.55 ^a^	0.011
Boarding	4367 (88.54)	90.23 ± 19.88		
Non-boarding	565 (11.46)	87.55 ± 21.38		
Father’s education attainment			94.28 ^b^	<0.001
Primary or below	138 (2.80)	79.93 ± 24.30		
Junior high school	1717 (34.81)	87.80 ± 20.19		
Senior high school	1680 (34.06)	89.71 ± 19.89		
College or higher	1397 (28.33)	93.77 ± 18.96		
Mother’s education attainment			94.44 ^b^	<0.001
Primary or below	263 (5.33)	80.88 ± 22.38		
Junior high school	1753 (35.54)	88.09 ± 20.33		
Senior high school	1531 (31.04)	90.61 ± 19.86		
College or higher	1385 (28.09)	93.19 ± 18.75		
Only child			−5.03 ^a^	<0.001
Multiple-child	3105 (62.96)	88.78 ± 20.30		
Only child	1827 (37.04)	91.86 ± 19.55		
Family economic status			296.17 ^b^	<0.001
Poor	211 (4.28)	75.20 ± 23.53		
Normal	2290 (46.43)	86.44 ± 19.33		
Good	2431 (49.29)	94.17 ± 19.17		
Region			3.85 ^a^	<0.001
Rural	1936 (39.25)	91.09 ± 20.37		
Urban	2996 (60.75)	89.17 ± 19.86		
Areas			4.58 ^a^	<0.001
Coastal	2658 (50.23)	91.13 ± 19.85		
Inland	2274 (49.77)	88.51 ± 20.26		

^a^ Z value, ^b^ H value.

**Table 2:** Association of parental healthy behaviors with proactive health behaviors among adolescents with and overweight and obesity.

	Model 1			Model 2			Model 3			Model 4		
	β	95% CI	*p*	β	95% CI	*p*	β	95% CI	*p*	β	95% CI	*p*
Father’s healthy behaviors	1.090	0.889, 1.291	<0.001	0.847	0.654, 1.039	<0.001	0.534	0.354, 0.715	<0.001	0.442	0.263, 0.620	<0.001
Mother’s healthy behaviors	0.845	0.629, 1.061	<0.001	0.641	0.434, 0.847	<0.001	0.500	0.307, 0.692	<0.001	0.525	0.336, 0.714	<0.001
Age				−1.545	−2.217, −0.873	<0.001	−1.382	−2.006, −0.757	<0.001	−1.206	−1.826, −0.586	<0.001
Gender				1.777	0.736, 2.818	0.001	1.146	0.178, 2.114	0.020	1.184	0.226, 2.142	0.015
Ethnicity				0.338	−3.466, 4.142	0.862	0.510	−3.025, 4.045	0.777	0.412	−3.063, 3.886	0.816
Accommodation				0.465	−1.149, 2.079	0.572	0.841	−0.659, 2.341	0.272	−0.154	−1.658, 1.350	0.841
Self-efficacy				0.735	0.665, 0.806	<0.001	0.379	0.308, 0.449	<0.001	0.354	0.285, 0.423	<0.001
Father educational attainment												
Junior high school				3.568	0.271, 6.866	0.034	2.502	−0.563, 5.567	0.110	1.505	−1.511, 4.521	0.328
Senior high school				3.510	0.101, 6.920	0.044	2.272	−0.897, 5.442	0.160	1.228	−1.890, 4.347	0.440
College or higher				4.649	1.074, 8.223	0.011	2.973	−0.351, 6.296	0.080	1.733	−1.541, 5.007	0.300
Mother educational attainment												
Junior high school				3.965	1.487, 6.442	0.002	2.562	0.258, 4.866	0.029	2.114	−0.154, 4.382	0.068
Senior high school				5.447	2.819, 8.075	<0.001	3.799	1.355, 6.243	0.002	3.298	0.888, 5.707	0.007
College or higher				5.132	2.320, 7.945	<0.001	2.516	−0.104, 5.136	0.060	1.971	−0.617, 4.558	0.135
Family cohesion							0.612	0.569, 0.655	<0.001	0.590	0.547, 0.632	<0.001
Only child										0.736	−0.265, 1.738	0.150
Family economic status												
normal										6.870	4.544, 9.195	<0.001
good										11.267	8.924, 13.609	<0.001
Region										−2.561	−3.540, −1.583	<0.001
Areas										−0.908	−1.890, 0.073	0.070

Note: Model 2 was adjusted for variables from the individual system; Model 3 was adjusted for variables from the individual system and parent–child system; Model 4 was adjusted variables from the individual system, parent–child system, and family system.

**Table 3:** Mediating effects of self-efficacy and family cohesion on the relationship between parental healthy behaviors and proactive healthy behaviors.

	**β**	**SE**	**LICI**	**ULCI**
**Total Effect**				
Fathers’ healthy behaviors→Proactive health behaviors	1.090	0.103	0.889	1.291
Mothers’ healthy behaviors→Proactive health behaviors	0.845	0.110	0.629	1.061
	**β**	**SE**	**LICI**	**ULCI**
**Direct Effect**				
Fathers’ healthy behaviors→Proactive health behaviors	0.553	0.092	0.373	0.734
Mothers’ healthy behaviors→Proactive health behaviors	0.498	0.098	0.305	0.690
Self-efficacy→Proactive health behaviors	0.383	0.036	0.313	0.453
Family cohesion→Proactive health behaviors	0.623	0.022	0.581	0.666
Fathers’ healthy behaviors→Self-efficacy	0.250	0.039	0.173	0.326
Mothers’ healthy behaviors→Self-efficacy	0.264	0.042	0.181	0.346
Fathers’ healthy behaviors→Family cohesion	0.707	0.064	0.582	0.833
Mothers’ healthy behaviors→Family cohesion	0.395	0.069	0.260	0.530
	**β**	**Boot SE**	**Boot LICI**	**Boot ULCI**
**Indirect Effect**				
Fathers’ healthy behaviors→Self-efficacy→Proactive health behaviors	0.096	0.004	0.011	0.027
Fathers’ healthy behaviors→Family cohesion→Proactive health behaviors	0.441	0.008	0.070	0.105
Mothers’ healthy behaviors→Self-efficacy→Proactive health behaviors	0.101	0.004	0.011	0.027
Mothers’ healthy behaviors→Family cohesion→Proactive health behaviors	0.246	0.008	0.031	0.061

## Data Availability

The data presented in this study are available on request from the corresponding author due to privacy and ethical reasons.

## Supplementary Materials

The following supporting information can be downloaded at: https://www.mdpi.com/article/10.3390/nu17213377/s1, Table S1: Healthy Behaviors Questionnaire; Table S2: Family Adaptability and Cohesion Evaluation Scales; Table S3: Proactive Health Behaviors Scale; Table S4: Gender-specific analysis: association between parental healthy behaviors and proactive health behaviors among adolescents with and overweight and obesity; Table S5: Region-specific analysis: association between parental healthy behaviors and proactive health behaviors among adolescents with and overweight and obesity; Table S6: BMI-specific analysis: association between parental healthy behaviors and proactive health behaviors; Table S7: Bivariate correlation among parental healthy lifestyle, self-efficacy, family cohesion and proactive healthy behaviors; Figure S1: The mediation model and hypotheses.
