# Supplementary material for: Intergenerational Transmission of Proactive Health Behaviors Among Adolescents with Overweight or Obesity: The Mediating Role of Self-Efficacy and Family Cohesion
Source: Nutrients. 2025 Oct 28;17(21):3377. doi: 10.3390/nu17213377 (PMC12610138; doi:10.3390/nu17213377)
Supplement: Supplementary file 1 [file nutrients-17-03377-s001.zip › nutrients-3934780-supplementary.pdf]

Table S1 Healthy Behaviors Questionnaire

Items	Never	1-3 times per week	4-6 times per week	Every day
The frequency of your father's walking.				
The frequency of your father's physical activity.				
The frequency of your father's fitness activities.				
The frequency of your father's fruit intake.				
The frequency of your father's vegetable intake.				
The frequency of your mother's walking.				
The frequency of your mother's physical activity.				
The frequency of your mother's fitness activities.				
The frequency of your mother's fruit intake.				
The frequency of your mother's vegetable intake.				
Does your father smoke?	Yes ( )		No ( )	
Does your father drink alcohol more than twice a week?	Yes ( )		No ( )	
Does your mother smoke?	Yes ( )		No ( )	
Does your mother drink alcohol more than twice a week?	Yes ( )		No ( )	

Table S2 Family Adaptability and Cohesion Evaluation Scales

Items	Never	Rarely	Sometimes	Often	Always
Family members ask each other for help.					
In times of crisis, we turn to each other for support.					
Family members feel closer to other family members than to people outside the family.					
We togetherness as a family.					
We share interests and hobbies with each other.					
Family members consult other family members on their decisions.					
We approve of each other's friends.					
We feel closer to people outside the family than to other family members.					
We are supportive of each other during difficult times.					
We like to spend our free time with each other.					
Family members get along well with each other.					
Family members go along with what the family has decided to do.					
We confide in each other.					
We manage our free time independently of each other.					
We are able to depend on each other for things.					
We know each other's close friends.					

Table S3 Proactive Health Behaviors Scale.

Dimensions	Items	Always	Often	Sometimes	Rarely	Never
Health Responsibility	I proactively acquire knowledge and information about health manage-ment.					
	I will positively attend programs about preventing health issues and improving my health.					
	I can implement my health plan in my daily life.					
	I proactively seek health guidance, counselling or assistance for my health.					
	I will timely report unusual symptoms to someone such as parents, teachers or school doctor/nurse.					
Exercise	I will engage in moderate intensity physical activity per week.					
	I will participate in leisure activities or sports proactively.					
	I will walk or do something actively during my free time.					
	I will invite friends to exercise with me.					
	I will invite my family to exercise with me.					
Diet	Eat three regular meals each day.					
	Eat vegetables and fruits every day.					
	Actively eat fish, poultry, meat, eggs and other high-quality protein foods.					
	Intake enough milk and dairy products every day.					
	I will check the nutrition facts labels on food packages.					
	I will avoid fried foods.					
Mental health	I will take time to relax each day					
	I will take time for myself to do something I like					
	I will take the initiative to discuss my					

	problems with those around me and try to solve them (problems that affect me mentally and psychologically)					
	I will take active action to relieve stress (such as communicating with friends, seeking help from family members).					
Self-discipline	I will discourage family members or friends from smoking or drinking					
	I will avoid smoking and drinking					
	I will sleep 8-9 hours each night.					
	I will reduce exposure to electronic screens.					

Table S4 Gender-specific analysis: association between parental healthy behaviors and proactive health behaviors among adolescents with and overweight and obesity.

	Female			Male		
	β	95% CI	P	β	95% CI	P
Father's healthy behaviors	0.284	0.002,0.567	0.049	0.551	0.321,0.781	<0.001
Mother's healthy behaviors	0.758	0.457,1.059	<0.001	0.380	0.137,0.624	0.002
Age	-0.109	-0.758,0.540	0.742	0.079	-0.454,0.612	0.771
Ethnicity	0.953	-4.160,6.066	0.715	-0.116	-4.849,4.617	0.962
Accommodation	0.597	-1.757,2.950	0.619	-0.999	-2.957,0.958	0.317
Self-efficacy	0.422	0.308,0.536	<0.001	0.307	0.219,0.394	<0.001
Father educational attainment						
Junior high school	5.162	0.246,10.078	0.040	-0.132	-3.968,3.705	0.946
Senior high school	4.052	-1.042,9.145	0.119	0.203	-3.756,4.162	0.920
College or higher	4.704	-0.655,10.064	0.085	0.540	-3.610,4.690	0.799
Mother educational attainment						
Junior high school	4.046	0.415,7.676	0.029	1.086	-1.833,4.006	0.466
Senior high school	4.869	0.971,8.766	0.014	2.422	-0.662,5.505	0.124
College or higher	4.140	-0.040,8.320	0.052	0.759	-2.553,4.072	0.653
Family cohesion	0.529	0.465,0.593	<0.001	0.636	0.579,0.692	<0.001
Only child	-0.120	-1.782,1.543	0.888	1.338	0.075,2.600	0.038
Family economic status						
normal	7.503	3.898,11.108	<0.001	6.054	3.007,9.101	<0.001
good	10.975	7.328,14.622	<0.001	11.073	8.008,14.137	<0.001
Region	-2.650	-4.204,-1.097	0.001	-2.604	-3.866,-1.341	<0.001
Areas	-1.836	-3.406,-0.267	0.022	-0.589	-1.845,0.667	0.358

Table S5. Region-specific analysis: association between parental healthy behaviors and proactive health behaviors among adolescents with and overweight and obesity.

	Rural			Urban		
	β	95% CI	P	β	95% CI	P
Father's healthy behaviors	0.431	0.128,0.735	0.005	0.481	0.261,0.700	<0.001
Mother's healthy behaviors	0.281	-0.035,0.598	0.082	0.708	0.473,0.943	<0.001
Age	0.050	-0.634,0.734	0.886	0.002	-0.515,0.518	0.994
Gender	0.764	-0.802,2.329	0.339	1.193	-0.016,2.402	0.053
Ethnicity	-1.668	-8.453,5.116	0.630	1.063	-2.955,5.081	0.604
Accommodation	-1.417	-3.365,0.530	0.154	1.882	-0.557,4.322	0.130
Self-efficacy	0.264	0.155,0.373	<0.001	0.434	0.345,0.524	<0.001
Father educational attainment						
Junior high school	0.409	-4.256,5.075	0.863	2.157	-1.801,6.115	0.285
Senior high school	0.465	-4.418,5.349	0.852	1.676	-2.372,5.723	0.417
College or higher	0.083	-5.222,5.387	0.976	2.493	-1.694,6.680	0.243
Mother educational attainment						
Junior high school	2.620	-0.708,5.949	0.123	1.775	-1.354,4.905	0.266
Senior high school	4.661	1.026,8.296	0.012	2.591	-0.659,5.840	0.118
College or higher	1.966	-2.142,6.075	0.348	2.171	-1.232,5.574	0.211
Family cohesion	0.621	0.551,0.691	<0.001	0.575	0.522,0.629	<0.001
Only child	0.492	-1.152,2.137	0.557	1.054	-0.215,2.323	0.104
Family economic status						
normal	9.912	6.401,13.422	<0.001	3.555	0.437,6.673	0.025
good	17.284	13.762,20.806	<0.001	5.984	2.834,9.135	<0.001
Areas	-1.328	-2.981,0.325	0.115	-0.646	-1.906,0.615	0.315

Table S6. BMI-specific analysis: association between parental healthy behaviors and proactive health behaviors.

	Underweight and normal			Overweight and obesity		
	β	95% CI	P	β	95% CI	P
Father's healthy behaviors	0.311	0.165,0.456	<0.001	0.442	0.263,0.620	<0.001
Mother's healthy behaviors	0.723	0.567,0.880	<0.001	0.525	0.336,0.714	<0.001
Age	0.352	0.001,0.704	0.049	-1.206	-1.826,-0.586	<0.001
Gender	1.175	0.417,1.932	0.002	1.184	0.226,2.142	0.015
Ethnicity	-0.899	-3.799,2.000	0.543	0.412	-3.063,3.886	0.816
Accommodation	-0.516	-1.646,0.615	0.371	-0.154	-1.658,1.350	0.841
Self-efficacy	0.420	0.362,0.478	<0.001	0.354	0.285,0.423	<0.001
Father educational attainment						
Junior high school	-0.765	-2.941,1.411	0.491	1.505	-1.511,4.521	0.328
Senior high school	0.402	-1.877,2.681	0.729	1.228	-1.890,4.347	0.440
College or higher	-0.437	-2.861,1.988	0.724	1.733	-1.541,5.007	0.300
Mother educational attainment						
Junior high school	0.250	-1.540,2.040	0.784	2.114	-0.154,4.382	0.068
Senior high school	0.353	-1.565,2.272	0.718	3.298	0.888,5.707	0.007
College or higher	-0.362	-2.445,1.721	0.733	1.971	-0.617,4.558	0.135
Family cohesion	0.623	0.588,0.658	<0.001	0.590	0.547,0.632	<0.001
Only child	0.662	-0.209,1.534	0.136	0.736	-0.265,1.738	0.150
Family economic status						
normal	2.137	0.272,4.002	0.025	6.870	4.544,9.195	<0.001
good	5.669	3.783,7.556	<0.001	11.267	8.924,13.609	<0.001
Region	-1.326	-2.151,-0.501	0.002	-2.561	-3.540,-1.583	<0.001
Areas	-1.766	-2.314,-1.218	<0.001	-0.908	-1.890,0.073	0.070

Table S7 Bivariate correlation among parental healthy lifestyle, self-efficacy, family cohesion and proactive healthy behaviors

Variable	Correlation matrix				
	1	2	3	4	5
1.Fathers' healthy lifestyle	1.000				
2.Mothers' healthy lifestyle	0.733***	1.000			
3.Self-efficacy	0.228***	0.230***	1.000		
4.Family cohesion	0.314***	0.290***	0.358***	1.000	
5.Proactive health behaviors	0.335***	0.330***	0.379***	0.506***	1.000

***P<0.001

Figure S1

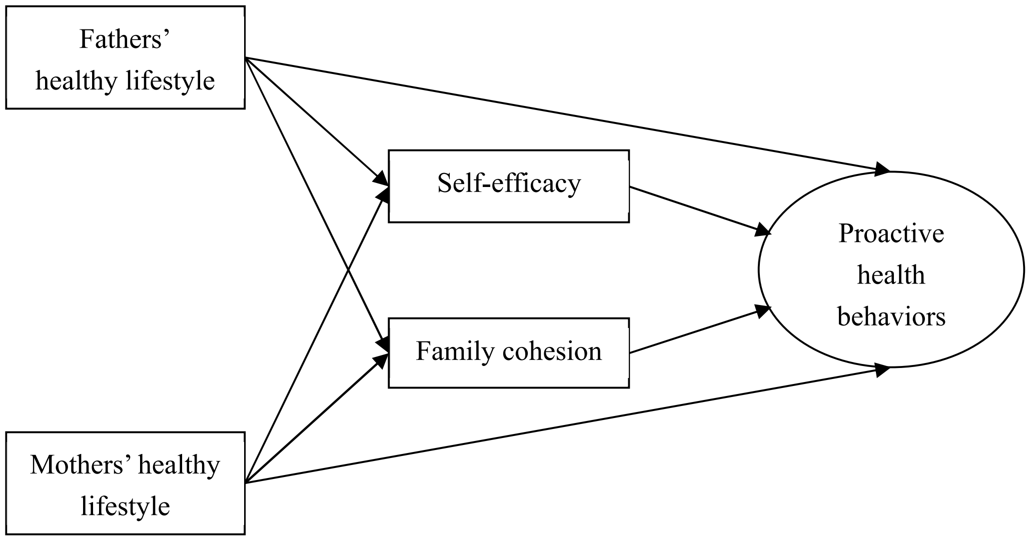

Figure S1. The mediation model and hypotheses.
